# *catena*-Poly[[[bis­(thio­cyanato-κ*N*)zinc(II)]-μ-1,2-bis­{[2-(2-pyrid­yl)-1*H*-imidazol-1-yl]meth­yl}benzene] 0.28-hydrate]

**DOI:** 10.1107/S1600536810027571

**Published:** 2010-07-17

**Authors:** Fei Han, Haochen Shi, Yunfeng Gao, Hongjun Ma

**Affiliations:** a Department of Physics Education, Changchun Normal University, 667 Changji Highway (North), Erdao District, Jilin Province 130032, People’s Republic of China

## Abstract

The title one-dimensional coordination polymer, {[Zn(NCS)_2_(C_24_H_20_N_6_)_2_]·0.28H_2_O}_*n*_, was obtained by the reaction of Zn(OAc)_2_·2H_2_O, KSCN and 1,2-bis­{[2-(2-pyrid­yl)-1*H*-imid­azol-1-yl]meth­yl}benzene (hereafter *L*). The Zn^II^ ion shows a distorted octa­hedral coordination geometry and is coordin­ated by two N atoms from two SCN^−^ anions and four N atoms from two organic ligands. The *L* ligands act as bridging bis-chelating ligands with *cis* coordination modes at the Zn^II^ ion. One-dimensional coordination polymers are arranged into layers by π–π stacking inter­actions between the imidazole rings of adjacent chains, with an inter­planar distance of 3.46 (1) Å and centroid–centroid distances of 3.8775 (16) Å. One of the thio­cyanate ligands is disordered over two positions with an occupancy factor of 0.564 (3) for the major component. The partially occupied water mol­ecule forms an O—H⋯S hydrogen bond with the disordered thio­cyanate group.

## Related literature

For backgroud to the topologies, supra­molecular structures and applications of metal-organic frameworks (MOFs), see: Dybtsev *et al.* (2004 ▶); Evans & Lin (2002 ▶); Moulton & Zaworotko (2001 ▶). For coordination modes of organic ligands, see: Janiak (2003 ▶). For similar structures, see: Dai *et al.* (2002 ▶); Luan *et al.* (2006 ▶). For the synthesis of 1,2-bis­{[2-(2-pyrid­yl)-1*H*-imidazol-1-yl]meth­yl}benzene, see: Li *et al.* (2008 ▶). 
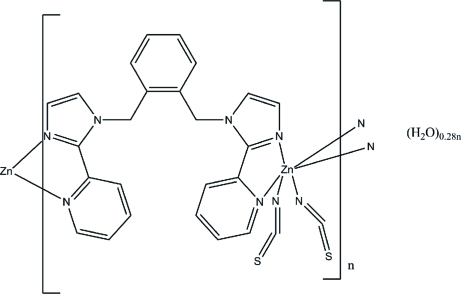


## Experimental

### 

#### Crystal data

[Zn(NCS)_2_(C_24_H_20_N_6_)_2_]·0.28H_2_O
*M* *_r_* = 579.03
Monoclinic, 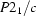

*a* = 7.8780 (4) Å
*b* = 13.1770 (7) Å
*c* = 25.9620 (14) Å
β = 98.462 (1)°
*V* = 2665.7 (2) Å^3^
*Z* = 4
Mo *K*α radiation
μ = 1.11 mm^−1^
*T* = 293 K
0.26 × 0.22 × 0.21 mm

#### Data collection

Bruker APEX CCD area-detector diffractometer
Absorption correction: multi-scan (*SADABS*; Sheldrick, 1996 ▶) *T*_min_ = 0.750, *T*_max_ = 0.792
13328 measured reflections
4707 independent reflections
3127 reflections with *I* > 2σ(*I*)
*R*_int_ = 0.036

#### Refinement

*R*[*F* ^2^ > 2σ(*F* ^2^)] = 0.038
*wR*(*F* ^2^) = 0.102
*S* = 1.04
4707 reflections
362 parameters
30 restraints
H-atom parameters constrained
Δρ_max_ = 0.38 e Å^−3^
Δρ_min_ = −0.33 e Å^−3^

### 

Data collection: *SMART* (Bruker, 1997 ▶); cell refinement: *SAINT* (Bruker, 1997 ▶); data reduction: *SAINT*; program(s) used to solve structure: *SHELXS97* (Sheldrick, 2008 ▶); program(s) used to refine structure: *SHELXL97* (Sheldrick, 2008 ▶); molecular graphics: *DIAMOND* (Brandenburg & Putz, 2008 ▶); software used to prepare material for publication: *SHELXL97*.

## Supplementary Material

Crystal structure: contains datablocks I, global. DOI: 10.1107/S1600536810027571/gk2287sup1.cif

Structure factors: contains datablocks I. DOI: 10.1107/S1600536810027571/gk2287Isup2.hkl

Additional supplementary materials:  crystallographic information; 3D view; checkCIF report

## Figures and Tables

**Table 1:** Hydrogen-bond geometry (Å, °)

*D*—H⋯*A*	*D*—H	H⋯*A*	*D*⋯*A*	*D*—H⋯*A*
O1*W*—H2*W*⋯S1^i^	0.85	2.68	3.30 (2)	132

Symmetry code: (i) 
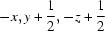
.

**Fig. 1.**
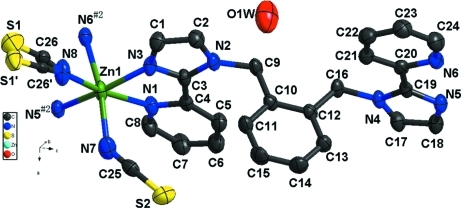
A displacement ellipsoids view of the title compound with the displacement ellipsoids drawn at the 30% probability level. Symmetry code #2: x, -y+1/2, z-1/2.

**Fig. 2.**
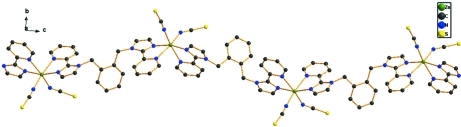
View of the one-dimensional chain.

**Fig. 3.**
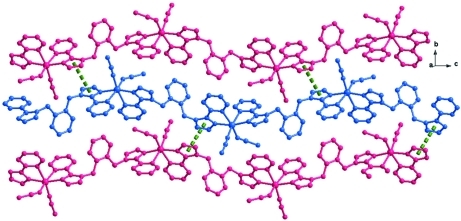
View of the two-dimensional supramolecular structure formed by π–π stacking interactions.

## supplementary crystallographic information

### Comment

In recent years, there is an increasing interest in metal-organic frameworks (MOFs) for the versatile architectures and intriguing topologies as well as their wide potential applications (Dybtsev *et al.* 2004; Evans & Lin, 2002). A universal strategy for the construction of MOFs is dependent primarily on the appropriate choice of inorganic building blocks and different organic ligands. Among them, N-donor organic ligands are important because of their divers coordination modes to metal ions resulting in different structures (Janiak, 2003) and the ability to form of weak interactions to assemble supramolecular structures (Moulton & Zaworotko, 2001). In this case, 1,2-bis{[2-(2-pyridyl)-1*H*-imidazol-1-yl]methyl}benzene (hereafter *L*) is selected as organic ligand and reacted with Zn(OAc)_2_.2H_2_O and KSCN to obtain the title compound.

In the title compound, there is one kind of *L* ligand, Zn^II^ ion and two kinds of SCN^-^ anions in the unit cell (Fig. 1). Each Zn^II^ ion is coordinated by two nitrogen atoms from two SCN^-^ anions and four aromatic N atoms from two different *L* molecules with normal Zn—N distances (Dai *et al.* 2002; Luan *et al.* 2006), showing a distorted octahedral coordination geometry. Each *L* molecule is acting as a bridging bis-bidentate ligand coordinated to two Zn^II^ ions to form polymeric one-dimensional chain (Fig. 2). Moreover, a two-dimensional supramolecular layer is finally formed by linking these chains through the π–π stacking interactions between imidazole rings from adjacent chains, with the plane to plane distance of 3.46 (1) Å and the centroid-centroid distances of 3.87 (8) Å. (Fig. 3).

### Experimental

A mixture of Zn(OAc)_2_. 2H_2_O (1 mmol), *L* (1 mmol) (Li *et al.* 2008), KSCN (0.10 g, 2 mmol) and H_2_O (8 ml) was sealed in a 18 ml Teflon- lined stainless steel container which was heated to 120 °C for 50 h, and cooled to room temperature. Colorless polyhedron crystals were collected in 85% yield.

### Refinement

The disordered SCN^-^ anion was refined with S and C atoms split over two sites, with the sum of the occupancy factors equal to 1.00. In this anion restraints were imposed on the anion geometry (DFIX instructions of SHELXL-97) and anisotropic displacement parameter of C and S atoms (ISOR instruction). The occupancy factor of the water molecule was initially refined but it was fixed in the final refinement cycles. Positions of H atoms from water molecules were calculated assuming interactions with the anion S atoms and these atoms were refined as riding with O-H = 0.85 Å and *U*_iso_=1.5*U*_eq_ (O). All H atoms bound to C atoms were positioned geometrically and refined as riding atoms, with C—H = 0.93 and 0.97 Å, and *U*_iso_=1.2*U*_eq_ (C).

### Crystal data

**Table:**

[Zn(NCS)_2_(C_24_H_20_N_6_)_2_]·0.28H_2_O	*F*(000) = 1187
*M**_r_* = 579.03	*D*_x_ = 1.443 Mg m^−^^3^
Monoclinic, *P*2_1_/*c*	Mo *K*α radiation, λ = 0.71069 Å
Hall symbol: -P 2ybc	Cell parameters from 2199 reflections
*a* = 7.8780 (4) Å	θ = 1.6–26.4°
*b* = 13.1770 (7) Å	µ = 1.11 mm^−^^1^
*c* = 25.9620 (14) Å	*T* = 293 K
β = 98.462 (1)°	Block, colorless
*V* = 2665.7 (2) Å^3^	0.26 × 0.22 × 0.21 mm
*Z* = 4	

### Data collection

**Table:**

Bruker APEX CCD area-detector diffractometer	4707 independent reflections
Radiation source: fine-focus sealed tube	3127 reflections with *I* > 2σ(*I*)
graphite	*R*_int_ = 0.036
ω scans	θ_max_ = 25.0°, θ_min_ = 1.6°
Absorption correction: multi-scan (*SADABS*; Sheldrick, 1996)	*h* = −9→9
*T*_min_ = 0.750, *T*_max_ = 0.792	*k* = −13→15
13328 measured reflections	*l* = −28→30

### Refinement

**Table:**

Refinement on *F*^2^	Primary atom site location: structure-invariant direct methods
Least-squares matrix: full	Secondary atom site location: difference Fourier map
*R*[*F*^2^ > 2σ(*F*^2^)] = 0.038	Hydrogen site location: inferred from neighbouring sites
*wR*(*F*^2^) = 0.102	H-atom parameters constrained
*S* = 1.04	*w* = 1/[σ^2^(*F*_o_^2^) + (0.0472*P*)^2^ + 0.0052*P*] where *P* = (*F*_o_^2^ + 2*F*_c_^2^)/3
4707 reflections	(Δ/σ)_max_ = 0.001
362 parameters	Δρ_max_ = 0.38 e Å^−^^3^
30 restraints	Δρ_min_ = −0.33 e Å^−^^3^

### Special details

**Table:**

Geometry. All esds (except the esd in the dihedral angle between two l.s. planes) are estimated using the full covariance matrix. The cell esds are taken into account individually in the estimation of esds in distances, angles and torsion angles; correlations between esds in cell parameters are only used when they are defined by crystal symmetry. An approximate (isotropic) treatment of cell esds is used for estimating esds involving l.s. planes.
Refinement. Refinement of F^2^ against ALL reflections. The weighted R-factor wR and goodness of fit S are based on F^2^, conventional R-factors R are based on F, with F set to zero for negative F^2^. The threshold expression of F^2^ > 2sigma(F^2^) is used only for calculating R-factors(gt) etc. and is not relevant to the choice of reflections for refinement. R-factors based on F^2^ are statistically about twice as large as those based on F, and R- factors based on ALL data will be even larger.

### Fractional atomic coordinates and isotropic or equivalent isotropic displacement parameters (Å^2^)

**Table:**

	*x*	*y*	*z*	*U*_iso_*/*U*_eq_	Occ. (<1)
C1	0.1405 (4)	0.0988 (2)	0.23131 (12)	0.0627 (9)	
H1	0.0560	0.0583	0.2128	0.075*	
C2	0.1380 (4)	0.1355 (2)	0.27956 (12)	0.0628 (9)	
H2	0.0527	0.1253	0.3003	0.075*	
C3	0.3735 (4)	0.1861 (2)	0.25115 (10)	0.0498 (7)	
C4	0.5346 (4)	0.2347 (2)	0.24331 (11)	0.0488 (7)	
C5	0.6518 (4)	0.2810 (2)	0.28088 (12)	0.0652 (9)	
H5	0.6360	0.2801	0.3157	0.078*	
C6	0.7924 (5)	0.3283 (3)	0.26590 (14)	0.0787 (10)	
H6	0.8719	0.3607	0.2905	0.094*	
C7	0.8146 (4)	0.3274 (3)	0.21460 (15)	0.0797 (11)	
H7	0.9059	0.3615	0.2034	0.096*	
C8	0.6981 (4)	0.2748 (3)	0.18015 (13)	0.0715 (10)	
H8	0.7162	0.2710	0.1456	0.086*	
C9	0.3326 (4)	0.2402 (2)	0.34290 (10)	0.0562 (8)	
H9A	0.2287	0.2595	0.3564	0.067*	
H9B	0.3960	0.3018	0.3382	0.067*	
C10	0.4402 (4)	0.1734 (2)	0.38252 (10)	0.0493 (7)	
C11	0.4785 (4)	0.0736 (2)	0.37134 (12)	0.0633 (9)	
H11	0.4402	0.0475	0.3384	0.076*	
C12	0.4998 (3)	0.2120 (2)	0.43188 (10)	0.0464 (7)	
C13	0.5948 (4)	0.1497 (2)	0.46849 (11)	0.0574 (8)	
H13	0.6355	0.1751	0.5014	0.069*	
C14	0.6294 (4)	0.0511 (3)	0.45663 (13)	0.0699 (9)	
H14	0.6921	0.0100	0.4816	0.084*	
C15	0.5719 (4)	0.0128 (3)	0.40811 (14)	0.0747 (10)	
H15	0.5961	−0.0539	0.4001	0.090*	
C16	0.4598 (4)	0.3198 (2)	0.44434 (10)	0.0514 (7)	
H16A	0.5126	0.3644	0.4215	0.062*	
H16B	0.3366	0.3297	0.4368	0.062*	
C17	0.6633 (4)	0.4061 (2)	0.51368 (11)	0.0594 (8)	
H17	0.7419	0.4278	0.4926	0.071*	
C18	0.6701 (4)	0.4240 (2)	0.56517 (12)	0.0610 (8)	
H18	0.7552	0.4611	0.5857	0.073*	
C19	0.4434 (4)	0.3341 (2)	0.54193 (10)	0.0460 (7)	
C20	0.2902 (4)	0.2752 (2)	0.54843 (10)	0.0480 (7)	
C21	0.1991 (4)	0.2117 (2)	0.51218 (11)	0.0554 (8)	
H21	0.2308	0.2040	0.4793	0.066*	
C22	0.0594 (4)	0.1596 (3)	0.52576 (13)	0.0691 (9)	
H22	−0.0047	0.1173	0.5017	0.083*	
C23	0.0159 (4)	0.1703 (3)	0.57424 (14)	0.0728 (10)	
H23	−0.0774	0.1357	0.5839	0.087*	
C24	0.1140 (4)	0.2337 (3)	0.60837 (13)	0.0715 (10)	
H24	0.0857	0.2404	0.6417	0.086*	
N1	0.5617 (3)	0.22928 (18)	0.19344 (9)	0.0557 (6)	
N2	0.2857 (3)	0.19105 (18)	0.29238 (8)	0.0531 (6)	
N3	0.2880 (3)	0.13082 (18)	0.21386 (9)	0.0556 (6)	
N4	0.5181 (3)	0.34985 (17)	0.49856 (8)	0.0490 (6)	
N5	0.5331 (3)	0.37948 (18)	0.58233 (9)	0.0525 (6)	
N6	0.2472 (3)	0.28627 (19)	0.59660 (9)	0.0566 (7)	
N7	0.6024 (4)	0.0045 (2)	0.18550 (12)	0.0833 (9)	
C25	0.6692 (4)	−0.0180 (2)	0.22631 (14)	0.0603 (8)	
S2	0.75961 (13)	−0.04934 (9)	0.28408 (4)	0.0927 (3)	
N8	0.2453 (4)	−0.0111 (2)	0.11975 (10)	0.0852 (10)	
C26	0.1381 (15)	−0.0639 (10)	0.1031 (6)	0.052 (3)	0.56 (3)
S1	0.0107 (12)	−0.1515 (10)	0.0796 (4)	0.1109 (18)	0.56 (3)
C26'	0.1698 (19)	−0.0857 (8)	0.1087 (8)	0.042 (3)	0.44 (3)
S1'	0.0605 (18)	−0.1874 (10)	0.0912 (4)	0.091 (3)	0.44 (3)
Zn1	0.41177 (5)	0.10351 (3)	0.149130 (12)	0.05821 (16)	
O1W	−0.0298 (16)	0.1050 (10)	0.3898 (4)	0.154 (5)	0.28
H1W	−0.1226	0.0745	0.3782	0.231*	0.28
H2W	−0.0408	0.1642	0.3768	0.231*	0.28

### Atomic displacement parameters (Å^2^)

**Table:**

	*U*^11^	*U*^22^	*U*^33^	*U*^12^	*U*^13^	*U*^23^
C1	0.070 (2)	0.072 (2)	0.0484 (19)	−0.0175 (18)	0.0148 (17)	−0.0006 (16)
C2	0.063 (2)	0.080 (2)	0.050 (2)	−0.0060 (18)	0.0228 (17)	0.0030 (16)
C3	0.0625 (19)	0.0526 (18)	0.0363 (16)	−0.0014 (15)	0.0143 (15)	−0.0020 (13)
C4	0.0609 (19)	0.0455 (17)	0.0416 (17)	0.0010 (15)	0.0127 (15)	−0.0043 (13)
C5	0.072 (2)	0.069 (2)	0.055 (2)	−0.0104 (19)	0.0133 (18)	−0.0107 (17)
C6	0.076 (2)	0.085 (3)	0.075 (3)	−0.014 (2)	0.010 (2)	−0.024 (2)
C7	0.072 (2)	0.087 (3)	0.087 (3)	−0.027 (2)	0.034 (2)	−0.018 (2)
C8	0.081 (2)	0.077 (2)	0.064 (2)	−0.015 (2)	0.037 (2)	−0.0131 (18)
C9	0.077 (2)	0.0581 (19)	0.0361 (16)	0.0103 (17)	0.0170 (15)	−0.0024 (14)
C10	0.0592 (18)	0.0525 (19)	0.0394 (17)	0.0047 (15)	0.0185 (14)	0.0039 (13)
C11	0.083 (2)	0.056 (2)	0.0514 (19)	0.0117 (18)	0.0113 (17)	−0.0073 (15)
C12	0.0553 (17)	0.0495 (18)	0.0379 (16)	0.0036 (14)	0.0185 (14)	0.0064 (13)
C13	0.069 (2)	0.058 (2)	0.0458 (18)	0.0074 (17)	0.0093 (16)	0.0041 (15)
C14	0.078 (2)	0.062 (2)	0.068 (2)	0.0175 (19)	0.0052 (19)	0.0107 (18)
C15	0.094 (3)	0.054 (2)	0.077 (2)	0.023 (2)	0.013 (2)	−0.0041 (19)
C16	0.073 (2)	0.0531 (18)	0.0303 (15)	−0.0011 (16)	0.0139 (14)	0.0033 (13)
C17	0.075 (2)	0.061 (2)	0.0472 (19)	−0.0101 (17)	0.0248 (17)	−0.0008 (15)
C18	0.078 (2)	0.059 (2)	0.0481 (19)	−0.0122 (17)	0.0143 (17)	−0.0020 (15)
C19	0.0625 (19)	0.0425 (17)	0.0356 (16)	0.0050 (15)	0.0156 (15)	0.0051 (13)
C20	0.0558 (18)	0.0485 (17)	0.0406 (17)	0.0087 (15)	0.0102 (14)	0.0085 (13)
C21	0.0595 (19)	0.063 (2)	0.0448 (18)	0.0008 (17)	0.0104 (15)	0.0038 (15)
C22	0.059 (2)	0.078 (2)	0.069 (2)	−0.0022 (19)	0.0041 (18)	−0.0009 (19)
C23	0.063 (2)	0.089 (3)	0.071 (2)	−0.008 (2)	0.0235 (19)	0.009 (2)
C24	0.074 (2)	0.088 (3)	0.057 (2)	−0.002 (2)	0.0279 (19)	0.0067 (19)
N1	0.0656 (16)	0.0576 (16)	0.0482 (15)	−0.0092 (13)	0.0228 (13)	−0.0068 (12)
N2	0.0680 (17)	0.0611 (16)	0.0325 (13)	0.0014 (14)	0.0155 (12)	−0.0008 (11)
N3	0.0691 (16)	0.0614 (16)	0.0382 (14)	−0.0151 (14)	0.0142 (13)	−0.0044 (12)
N4	0.0662 (16)	0.0491 (14)	0.0336 (13)	−0.0013 (13)	0.0140 (12)	0.0002 (11)
N5	0.0698 (16)	0.0537 (15)	0.0357 (14)	−0.0045 (13)	0.0136 (13)	0.0005 (11)
N6	0.0664 (17)	0.0643 (17)	0.0419 (14)	0.0053 (14)	0.0180 (13)	0.0081 (12)
N7	0.124 (3)	0.0640 (19)	0.0637 (19)	0.0083 (18)	0.0209 (18)	0.0063 (16)
C25	0.067 (2)	0.0435 (18)	0.075 (2)	0.0036 (16)	0.0286 (19)	−0.0005 (17)
S2	0.0803 (7)	0.1023 (8)	0.0907 (8)	0.0078 (6)	−0.0037 (6)	0.0145 (6)
N8	0.125 (3)	0.077 (2)	0.0556 (19)	−0.029 (2)	0.0210 (18)	−0.0141 (15)
C26	0.049 (5)	0.060 (5)	0.046 (5)	0.014 (4)	0.006 (4)	0.001 (4)
S1	0.065 (2)	0.129 (4)	0.137 (3)	−0.031 (3)	0.007 (2)	−0.033 (3)
C26'	0.038 (5)	0.053 (5)	0.034 (5)	0.020 (5)	0.006 (4)	0.001 (4)
S1'	0.072 (3)	0.113 (4)	0.089 (3)	−0.031 (3)	0.019 (2)	−0.043 (2)
Zn1	0.0859 (3)	0.0552 (2)	0.0370 (2)	−0.00799 (19)	0.02056 (19)	−0.00498 (16)
O1W	0.159 (11)	0.198 (14)	0.117 (10)	−0.065 (9)	0.058 (8)	−0.028 (8)

### Geometric parameters (Å, °)

**Table:**

C1—C2	1.346 (4)	C16—H16A	0.9700
C1—N3	1.374 (4)	C16—H16B	0.9700
C1—H1	0.9300	C17—C18	1.351 (4)
C2—N2	1.373 (4)	C17—N4	1.371 (4)
C2—H2	0.9300	C17—H17	0.9300
C3—N3	1.315 (3)	C18—N5	1.360 (4)
C3—N2	1.359 (3)	C18—H18	0.9300
C3—C4	1.463 (4)	C19—N5	1.319 (3)
C4—N1	1.345 (3)	C19—N4	1.361 (3)
C4—C5	1.382 (4)	C19—C20	1.465 (4)
C5—C6	1.376 (4)	C20—N6	1.351 (3)
C5—H5	0.9300	C20—C21	1.379 (4)
C6—C7	1.369 (4)	C21—C22	1.386 (4)
C6—H6	0.9300	C21—H21	0.9300
C7—C8	1.372 (4)	C22—C23	1.360 (4)
C7—H7	0.9300	C22—H22	0.9300
C8—N1	1.321 (4)	C23—C24	1.371 (4)
C8—H8	0.9300	C23—H23	0.9300
C9—N2	1.461 (3)	C24—N6	1.330 (4)
C9—C10	1.515 (4)	C24—H24	0.9300
C9—H9A	0.9700	N1—Zn1	2.250 (2)
C9—H9B	0.9700	N3—Zn1	2.095 (2)
C10—C11	1.390 (4)	N5—Zn1^i^	2.112 (2)
C10—C12	1.394 (4)	N6—Zn1^i^	2.265 (2)
C11—C15	1.374 (4)	N7—C25	1.151 (4)
C11—H11	0.9300	N7—Zn1	2.105 (3)
C12—C13	1.389 (4)	C25—S2	1.616 (4)
C12—C16	1.501 (4)	N8—C26	1.130 (4)
C13—C14	1.371 (4)	N8—Zn1	2.071 (3)
C13—H13	0.9300	C26—S1	1.591 (4)
C14—C15	1.371 (4)	C26'—S1'	1.621 (4)
C14—H14	0.9300	O1W—H1W	0.8500
C15—H15	0.9300	O1W—H2W	0.8500
C16—N4	1.469 (3)		
			
C2—C1—N3	109.0 (3)	C17—C18—H18	125.3
C2—C1—H1	125.5	N5—C18—H18	125.3
N3—C1—H1	125.5	N5—C19—N4	110.0 (2)
C1—C2—N2	106.8 (3)	N5—C19—C20	120.2 (2)
C1—C2—H2	126.6	N4—C19—C20	129.8 (3)
N2—C2—H2	126.6	N6—C20—C21	121.4 (3)
N3—C3—N2	110.0 (3)	N6—C20—C19	111.8 (2)
N3—C3—C4	120.1 (2)	C21—C20—C19	126.7 (3)
N2—C3—C4	129.9 (3)	C20—C21—C22	118.7 (3)
N1—C4—C5	121.3 (3)	C20—C21—H21	120.7
N1—C4—C3	112.0 (2)	C22—C21—H21	120.7
C5—C4—C3	126.8 (3)	C23—C22—C21	120.1 (3)
C6—C5—C4	118.8 (3)	C23—C22—H22	119.9
C6—C5—H5	120.6	C21—C22—H22	119.9
C4—C5—H5	120.6	C22—C23—C24	117.8 (3)
C7—C6—C5	119.7 (3)	C22—C23—H23	121.1
C7—C6—H6	120.2	C24—C23—H23	121.1
C5—C6—H6	120.2	N6—C24—C23	123.9 (3)
C6—C7—C8	118.0 (3)	N6—C24—H24	118.0
C6—C7—H7	121.0	C23—C24—H24	118.0
C8—C7—H7	121.0	C8—N1—C4	118.5 (3)
N1—C8—C7	123.5 (3)	C8—N1—Zn1	126.0 (2)
N1—C8—H8	118.3	C4—N1—Zn1	112.71 (18)
C7—C8—H8	118.3	C3—N2—C2	107.1 (2)
N2—C9—C10	113.2 (2)	C3—N2—C9	129.6 (3)
N2—C9—H9A	108.9	C2—N2—C9	123.2 (2)
C10—C9—H9A	108.9	C3—N3—C1	107.1 (2)
N2—C9—H9B	108.9	C3—N3—Zn1	115.93 (19)
C10—C9—H9B	108.9	C1—N3—Zn1	136.7 (2)
H9A—C9—H9B	107.7	C19—N4—C17	106.9 (2)
C11—C10—C12	118.9 (3)	C19—N4—C16	129.7 (2)
C11—C10—C9	121.7 (3)	C17—N4—C16	123.4 (2)
C12—C10—C9	119.4 (3)	C19—N5—C18	107.1 (2)
C15—C11—C10	121.1 (3)	C19—N5—Zn1^i^	116.53 (19)
C15—C11—H11	119.4	C18—N5—Zn1^i^	134.4 (2)
C10—C11—H11	119.4	C24—N6—C20	118.0 (3)
C13—C12—C10	119.3 (3)	C24—N6—Zn1^i^	126.6 (2)
C13—C12—C16	121.4 (3)	C20—N6—Zn1^i^	115.3 (2)
C10—C12—C16	119.3 (2)	C25—N7—Zn1	140.6 (3)
C14—C13—C12	120.7 (3)	N7—C25—S2	178.9 (3)
C14—C13—H13	119.6	C26—N8—Zn1	170.9 (9)
C12—C13—H13	119.6	N8—C26—S1	170.8 (12)
C15—C14—C13	120.4 (3)	N8—Zn1—N3	94.38 (10)
C15—C14—H14	119.8	N8—Zn1—N7	94.87 (13)
C13—C14—H14	119.8	N3—Zn1—N7	97.60 (11)
C14—C15—C11	119.7 (3)	N8—Zn1—N5^ii^	96.53 (10)
C14—C15—H15	120.2	N3—Zn1—N5^ii^	163.98 (9)
C11—C15—H15	120.2	N7—Zn1—N5^ii^	93.14 (11)
N4—C16—C12	114.5 (2)	N8—Zn1—N1	168.99 (10)
N4—C16—H16A	108.6	N3—Zn1—N1	74.61 (9)
C12—C16—H16A	108.6	N7—Zn1—N1	86.62 (11)
N4—C16—H16B	108.6	N5^ii^—Zn1—N1	94.28 (9)
C12—C16—H16B	108.6	N8—Zn1—N6^ii^	88.30 (11)
H16A—C16—H16B	107.6	N3—Zn1—N6^ii^	94.60 (9)
C18—C17—N4	106.7 (3)	N7—Zn1—N6^ii^	167.12 (10)
C18—C17—H17	126.6	N5^ii^—Zn1—N6^ii^	74.07 (9)
N4—C17—H17	126.6	N1—Zn1—N6^ii^	92.65 (9)
C17—C18—N5	109.3 (3)	H1W—O1W—H2W	105.1

Symmetry codes: (i) *x*, −*y*+1/2, *z*+1/2; (ii) *x*, −*y*+1/2, *z*−1/2.

### Hydrogen-bond geometry (Å, °)

**Table:**

*D*—H···*A*	*D*—H	H···*A*	*D*···*A*	*D*—H···*A*
O1W—H2W···S1^iii^	0.85	2.68	3.30 (2)	132

Symmetry codes: (iii) −*x*, *y*+1/2, −*z*+1/2.

## References

[bb1] Brandenburg, K. & Putz, H. (2008). *DIAMOND* Crystal Impact GbR, Bonn, Germany.

[bb2] Bruker (1997). *SMART* Bruker AXS Inc., Madison, Wisconsin, USA.

[bb3] Bruker (1999). *SAINT* Bruker AXS Inc., Madison, Wisconsin, USA.

[bb4] Dai, J.-C., Wu, X.-T., Fu, Z.-Y., Cui, C.-P., Hu, S.-M., Du, W.-X., Wu, L.-M., Zhang, H.-H. & Sun, R.-Q. (2002). *Inorg. Chem.* **41**, 1391–1396. 10.1021/ic010794y 11896706

[bb5] Dybtsev, D. N., Chun, H., Yoon, S. H., Kim, D. & Kim, K. (2004). *J. Am. Chem. Soc.* **126**, 32–33. 10.1021/ja038678c 14709045

[bb6] Evans, O. R. & Lin, W. (2002). *Acc. Chem. Res.* **35**, 511–522. 10.1021/ar0001012 12118990

[bb7] Janiak, C. (2003). *Dalton Trans.* pp. 2781–2804.

[bb8] Li, S.-L., Lan, Y.-Q., Ma, J.-F., Fu, Y.-M., Yang, J., Ping, G.-J., Liu, J. & Su, Z.-M. (2008). *Cryst. Growth Des.* **8**, 1610–1616.

[bb9] Luan, X.-J., Cai, X.-H., Wang, Y.-Y., Li, D.-S., Wang, C.-J., Liu, P., Hu, H.-M., Shi, Q.-Z. & Peng, S.-M. (2006). *Chem. Eur. J.* **12**, 6281–6289. 10.1002/chem.200501559 16739156

[bb10] Moulton, B. & Zaworotko, M. (2001). *Chem. Rev.* **101**, 1629–1658. 10.1021/cr9900432 11709994

[bb11] Sheldrick, G. M. (1996). *SADABS* University of Göttingen, Germany.

[bb12] Sheldrick, G. M. (2008). *Acta Cryst.* A**64**, 112–122. 10.1107/S0108767307043930 18156677

